# Mathematical Approaches of Branching Morphogenesis

**DOI:** 10.3389/fgene.2018.00673

**Published:** 2018-12-21

**Authors:** Christine Lang, Lisa Conrad, Odyssé Michos

**Affiliations:** Department of Biosystems Science and Engineering, ETH Zürich, Basel, Switzerland

**Keywords:** branching morphogenesis, mathematical modelling, FGF10, lung, kidney

## Abstract

Many organs require a high surface to volume ratio to properly function. Lungs and kidneys, for example, achieve this by creating highly branched tubular structures during a developmental process called branching morphogenesis. The genes that control lung and kidney branching share a similar network structure that is based on ligand-receptor reciprocal signalling interactions between the epithelium and the surrounding mesenchyme. Nevertheless, the temporal and spatial development of the branched epithelial trees differs, resulting in organs of distinct shape and size. In the embryonic lung, branching morphogenesis highly depends on FGF10 signalling, whereas GDNF is the driving morphogen in the kidney. Knockout of *Fgf10* and *Gdnf* leads to lung and kidney agenesis, respectively. However, FGF10 plays a significant role during kidney branching and both the FGF10 and GDNF pathway converge on the transcription factors ETV4/5. Although the involved signalling proteins have been defined, the underlying mechanism that controls lung and kidney branching morphogenesis is still elusive. A wide range of modelling approaches exists that differ not only in the mathematical framework (e.g., stochastic or deterministic) but also in the spatial scale (e.g., cell or tissue level). Due to advancing imaging techniques, image-based modelling approaches have proven to be a valuable method for investigating the control of branching events with respect to organ-specific properties. Here, we review several mathematical models on lung and kidney branching morphogenesis and suggest that a ligand-receptor-based Turing model represents a potential candidate for a general but also adaptive mechanism to control branching morphogenesis during development.

## Introduction

Branching morphogenesis is a common developmental process by which arborized structures with a high surface-to-volume ratio are created. In vertebrate organ development, branching morphogenesis describes how an epithelial organ bud branches into its surrounding mesenchyme. Epithelial-mesenchymal interactions via ligand-receptor signalling are well-established regulators of developmental processes such as growth and patterning (Clément et al., 2012a; Perrimon et al., 2012). Yet, it remains poorly understood how morphogen signalling guides branching morphogenesis in a reproducible fashion, while also allowing for adaptation to environmental changes and how the use of common developmental principles results in organs of different shape, size and function.

Even though the signalling networks relevant to organogenesis have been defined and gene functions and interactions have been studied intensively, it remains unclear how macroscopic features of branched organs, including size, network topology and spatial patterning are encoded. Mathematical modelling has proven to be a valuable method for examining the impact of signalling interactions in developmental processes, such as branching morphogenesis. Consequently, a wide range of modelling approaches have been established that can differ not only in the mathematical framework but also in the spatial scale (Peters et al., 2018).

In the following, we will focus on the role of fibroblast growth factor (FGF) 10 during branching morphogenesis of the vertebrate kidney and the lung and discuss to which extent mathematical models can help our understanding of branching morphogenesis. Lung and kidney branching has been studied in model organisms other than the mouse and the rat and orthologs of the genes described here have similar roles in other species, but are not further discussed in the context of this mini-review (Böttcher and Niehrs, 2005; Moura et al., 2011; Sakiyama, 2003; Shifley et al., 2012).

## Mesenchymal-Epithelial Interactions Via Morphogen Signalling and Their Receptors

FGF10 is a morphogen belonging to the fibroblast growth factor family and plays important roles in both kidney and lung development. FGF10 signalling is essential for lung development and guides directional bud outgrowth, sustains progenitor cell fate and affects expression of genes involved in a variety of developmental processes (Bellusci et al., 1997; Min et al., 1998; Lü et al., 2005; El Agha et al., 2014, 2017). In mesenchyme-free cultures of lung buds, only FGF10 and FGF1 are sufficient to induce branching of the epithelium (Nogawa and Ito, 1995; Bellusci et al., 1997). *Fgf10^-/-^* mice display kidney dysgenesis with impaired ureteric bud (UB) development and medullary dysplasia (Ohuchi et al., 2000; Michos et al., 2010).

**FIGURE 1:**
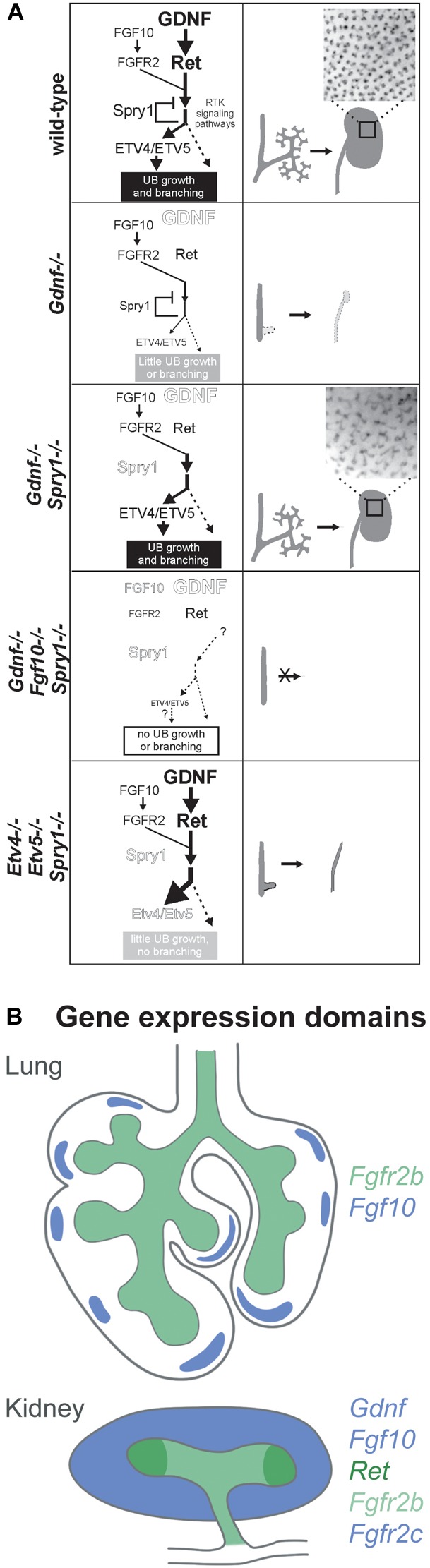
**(A)** Ureteric bud branching morphogenesis is cooperatively regulated by FGF10 and GDNF via *Etv4* and *Etv5*, whereas Sprouty1 acts as a negative regulator of FGF10 and GDNF signalling downstream of FGFR2 and RET. (1) Regulation of UB branching in the wild type: GDNF is the main promoting factor for UB outgrowth and branching, while FGF10 plays a minor role. Sprouty1 counteracts ligand-receptor signalling, resulting in the development of a normal kidney. (2) The absence of GDNF abrogates UB branching and UB outgrowth, resulting in kidney agenesis or severe hypodysplasia. (3) If both the negative regulator Sprouty1 and GDNF are absent FGF10 signalling is sufficient to drive *Etv4/Etv5* expression, allowing for UB branching and kidney development. The branching pattern differs from the wild type, which points to a GDNF specific regulation of UB morphogenesis. (4) In the triple knockout, there is not enough ligand-receptor signalling to drive UB outgrowth and branching, even though Sprouty1 is absent as well. (5) Increased receptor tyrosine kinase signalling resulting from the absence of Sprouty1 does not rescue the renal agenesis phenotype of *Etv4 -/- Etv5 -/-* mice. In this triple knockout, the UB grows out, but fails to undergo branching and only ureters develop, suggesting that branching morphogenesis is dependent on *Etv4* and *Etv5*. The insets in 1 and 3 show the pattern of UB tips on the surface of P0 wild type and double mutant kidneys. Reproduced with permission from Michos et al. (2010). **(B)** Gene expression domains of *Fgf10* and *Gdnf* and their receptors in the developing lung and kidney at E11.5. In the lung, *Fgf10* is expressed in the submesothelial mesenchyme in a spotty fashion opposed to growing buds. *Fgfr2b* is expressed in the lung epithelium. In the kidney, *Fgf10* and *Gdnf* are expressed in the metanephric mesenchyme. *Fgfr2* is expressed both in the mesenchyme and the epithelium, whereas *Ret* is restricted to the branch tips (ampulla). Expression domains are colour coded as indicated.

Glial cell line-derived neurotrophic factor (GDNF), a morphogen related to the TGF-beta growth factor family, is critical for kidney organogenesis. The absence of GDNF as well as of its receptor tyrosine kinase RET and co-receptor GFRα1 lead to kidney agenesis or severe hypodysplasia (Schuchardt et al., 1994, 1996; Moore et al., 1996; Pichel et al., 1996; Sánchez et al., 1996; Cacalano et al., 1998). Interestingly, both morphogens induce ERK signalling and converge on the transcription factors ETV4/5 to induce and promote branching (Lu et al., 2009; Michos et al., 2010). FGF10 rescues the *Gdnf^-/-^* phenotype in the absence of *Sprouty1*, an inhibitor of ERK signalling induced by FGF10 and GDNF, suggesting that FGF10 is an important regulator of branching during kidney development, which functions at least partly redundant to GDNF (Figure 1A) (Michos et al., 2010).

*Fgf10, Gdnf* and their receptors have distinct expression patterns during organogenesis (Figure 1B). In the lung, *Fgf10* is expressed in the submesothelial mesenchyme, so that FGF10 diffuses toward the epithelium to meet its receptor FGFR2b, which is expressed along the distal lung epithelium. Additionally, whole-mount *in situ* hybridisation of embryonic lungs has shown that *Fgf10* is expressed in spots “in front of” terminal end buds (Bellusci et al., 1997). Interestingly, both FGF10 and FGF7 bind FGFR2b, whereas only signalling via FGF10 results in receptor recycling and trafficking to the cell membrane, thereby increasing the amount of available FGFR2b (Francavilla et al., 2013). Besides the signalling function between mesenchymal and epithelial cells, a cell autonomous mode of function has been recently identified for FGF10 that is based on nuclear translocation within FGF10-producing cells (Mikolajczak et al., 2016).

During kidney development, *Fgf10* and *Gdnf* are expressed throughout the metanephric mesenchyme (MM) (Towers et al., 1998; Michos et al., 2010). *Ret* is expressed by tip cells that form the ampulla, a swelling of the terminal end buds of the UB epithelium. Cells that do not express or lose *Ret* expression are excluded from the ampulla (Shakya et al., 2005; Chi et al., 2009; Riccio et al., 2016). *Fgfr2* is expressed throughout the UB (Zhao et al., 2004; Sanna-Cherchi et al., 2013).

The expression patterns of ligands and receptors suggest that their spatial organisation plays an important role in guiding branching morphogenesis. However, studies in the lung have shown that uniform expression of *Fgf10* does not abolish branching and in the *Gdnf^-/-^; Sprouty1^-/-^* mutants UB branching occurs albeit both *Fgf10* and *Fgfr2* being expressed uniformly in the MM and the UB, respectively (Michos et al., 2010; Volckaert et al., 2013).

## Effect on the Epithelial Branching Pattern

Comparison of the branched epithelial trees of different organs could aid in understanding how organs of different shape and size are formed. Advancements in imaging techniques and analysis software are providing the opportunity to quantitatively study the morphometric differences between organs and to study the effect of mutations and drugs. To date, branching morphogenesis has been studied *ex vivo* using organ culture techniques that permit imaging of branching events over time or by reconstructing slices of fixed and stained organs or more recently by directly imaging dissected and fixed organs in 3D at different developmental time points (Michos, 2012).

The branching pattern of the lung has been well characterised. During the early stages of development, lateral branching dominates. From around E13.0 on, planar bifurcations subside as the main mode of branching and finally, orthogonal bifurcations are employed to efficiently fill all available space (Metzger et al., 2008). The same study reports lung branching to be extremely stereotyped, with only minor differences between mice of the same genetic background. Other publications have challenged this view, showing that branching varies after an initial, stereotypic establishment of the first branches and suggest that the epithelium can react to regional growth of the mesenchyme to efficiently fill the available space (Blanc et al., 2012; Short et al., 2013).

Kidney branching mainly employs terminal bifurcations and, more rarely, terminal trifurcations and lateral branching (Watanabe and Costantini, 2004; Short et al., 2014). *In vivo*, kidney branching does not follow stereotypic branching regimes on the organ-level, however, sub-trees that encompass daughter branches of the whole tree established by E12.5 seem to develop in a highly stereotypic manner (Short et al., 2014; Sampogna et al., 2015).

Absence of FGF10 or GDNF results in lung agenesis and kidney agenesis or severe hypodysplasia, respectively. Here, we focus on how the branching pattern is modified in response to changes in ligand-receptor signalling. FGF10 hypomorphic lungs exhibit reduced epithelial branching, resulting in lung hypoplasia (Ramasamy et al., 2007). This qualitative comparison of the branching pattern leaves open the question of whether branching is only temporarily delayed, or whether the morphology of the branched tree is changed as well. Elevated *Fgf10* expression in the pulmonary epithelium causes increased epithelial cell proliferation and progenitor state arrest, which leads to hyperplasia of the epithelium with large, empty lumens and larger interlobular distance (Nyeng et al., 2008).

The branching pattern of the developing kidney is modulated by the interplay between FGF10/GDNF signalling and negative regulation via SPRY1. Specific deletion of *Fgfr2* from the UB results in fewer UB tips with longer, thinner trunk segments (Zhao et al., 2004; Sims-Lucas et al., 2009). The absence of *Spry1* results in increased branching of the UB and the induction of supernumerary UBs, whereas *Gdnf^+/-^* kidneys show reduced branching (Basson et al., 2006). *Gdnf^-/-^;Spry1^-/-^* and *Ret^-/-^;Spry1^-/-^* kidneys show abnormal branching with irregular UB tip size, shape and branch angle (Michos et al., 2010).

Interestingly, gain-of-function mutations in *Fgfr2* cause major secondary branching defects in both lung and kidney that can be partly rescued by the genetic knockdown of Fgf10 expression (Hajihosseini et al., 2001, 2009).

## Impact of the Mesenchyme on Branching

Mesenchyme-free cultures of isolated lung buds and UBs have shown the ability of the epithelium to branch in the presence of the correct growth factors, which *in vivo* are expressed in the mesenchyme, showing an intrinsic capability of the epithelium to branch that does not depend on cell contacts of epithelium and mesenchyme (Nogawa and Ito, 1995; Bellusci et al., 1997; Qiao et al., 1999). These branched epithelial structures, however, lack the directionality and shape of the branched trees that result from branching morphogenesis of intact organs *in vivo* and in organ culture experiments, suggesting an important role of the mesenchyme in shaping the growing organ and in specifying the identity of epithelial cells.

As exemplified by FGF10 and GDNF in the context of lung and kidney, the growth factors expressed by the mesenchyme differ in their composition and spatiotemporal expression, suggesting that ligand-receptor signalling modulates the branching pattern. But differences in mechanical properties of the tissues and the extracellular matrix (ECM) could also have an impact. Tissue recombination experiments have been used to study epithelial-mesenchymal interactions and how the mesenchyme influences branching morphogenesis and cell fate specification of a branching epithelium (Grobstein, 1953, 1955; Saxen et al., 1976; Kispert et al., 1996; Iwai et al., 1998; Shannon et al., 1998; Ohtsuka et al., 2001; Lin et al., 2003).

Recombination of UB epithelium with lung mesenchyme at E11.5 results in a branching pattern more similar to that of an early lung (Lin et al., 2001). The lung mesenchyme also induces expression of surfactant protein C and changes the collagen pattern of the ECM to that of an embryonic lung, however, the epithelium continues to express UB-specific genes such as *Wnt11, Ret* and *Pax-2* (Kispert et al., 1996; Sainio et al., 1997; Lin et al., 2001).

## Modelling Branching Events as Stochastic Processes

There is a general distinction between approaches that model branching morphogenesis as a deterministic stereotypic programme of genetically encoded events and approaches that describe branching morphogenesis as a stochastic process based on generic rules (Miura, 2008; Wang et al., 2017).

Recently proposed stochastic models for kidney branching morphogenesis are based on rules regarding the ratio between epithelial growth speed and cell mobility (Hirashima et al., 2009a, 2017), the ratio between ureteric tip and mesenchymal tip cells (Zubkov et al., 2015), locally operative mechanism like inter tip-suppression (Lefevre et al., 2017), or a growth-factor dependent growth switch (Lambert et al., 2018). Although the models may explain why the structure of a branched tree evolves during development, these models are basically not addressing the underlying molecular regulatory processes of branching morphogenesis that would answer how the branching pattern forms.

Moreover, Hannezo et al. (2017) presented ‘A unifying theory of branching morphogenesis’ describing kidney branching as a self-organised process that is based on a simple set of statistical rules, including stochastic tip branching, random exploration of space and tip termination in high-density regions. However, a recent study is strongly challenging the stochastic nature of kidney branching morphogenesis and in particular the hypothesis that nephron differentiation leads to termination of tip branching (Short et al., 2018). Live imaging of cultured embryonic kidneys did not show any evidence for the influence of nephron formation on ureteric branching. Finally, Short et al. (2018) propose that kidney morphogenesis rather resembles stereotypic lung branching than stochastic mammary gland branching.

## Signalling Models Based on Diffusion-Limited Growth and Distance-Based Patterning

The signalling pathways controlling branching morphogenesis have been extensively studied and appear to play a key role in the regulation and formation of branched epithelial structures. Since the key signalling factors in lung and kidney branching morphogenesis are diffusible proteins and interact with their corresponding receptors, several deterministic reaction-diffusion models have been proposed to describe the branching behaviour. For lung branching morphogenesis, most of these models are based on diffusion-limited growth (Miura and Shiota, 2002; Hartmann and Miura, 2006, 2007), gradient-sensing mechanisms (Clément et al., 2012a,b), or distance-based patterning (Hirashima et al., 2009b), and have been already reviewed in detail (Miura, 2008; Iber and Menshykau, 2013). By using idealised 2D shapes of lung buds, these models describe the diffusion of FGF10 from the sub-mesothelial mesenchyme to the epithelium and propose a relationship between the distance of these two tissue layers and the branching modes. While a large distance leads to high FGF10 concentration at the tip, a thin mesenchyme will lead to a split FGF10 localization at the sides of the tip. Assuming that FGF10 triggers outgrowth, these FGF10 concentration profiles will result in bud elongation or bifurcation, respectively. The main limitation of these models is that they are not in agreement with the experimental observations that branching is still occurring under homogeneous *Fgf10* expression or in the absence of mesenchyme with FGF10 added to the medium (Nogawa and Ito, 1995; Volckaert et al., 2013).

## Geometry Effect and Image-Based Modelling

Information about the developmental process of morphogenesis is naturally image-based and branching patterns emerge on growing domains. Therefore, modelling morphogenesis is implicitly related to deforming shapes and domains and the question arises whether the geometry itself has an effect on patterning. It was computationally shown that the expression of ligands and receptors in different tissue layers gives rise to a diffusion-driven geometry effect (Nelson et al., 2006; Gleghorn et al., 2012). While this domain-specific ligand expression is able to create split branching patterns on static domains, it does not reproduce the actual outgrowth of buds on a growing domain because it is unstable under deforming curvature conditions (Menshykau et al., 2014).

George and Lubkin examined the effect of geometry on branch mode selection during lung development. Although the model identifies proximity and aspect ratios of the internal and external tissue surfaces as important geometric factors to determine branching modes like planar and orthogonal bifurcation, it is not able to explain lateral branching (George and Lubkin, 2018).

Considering the geometry effect, it is essential to perform model simulations on physiological growing domains in order to achieve biologically relevant predictions (Iber et al., 2015). The validity of most models was limited by the lack of high-resolution biological data both on the cellular and also on the whole-organ scale. Due to advancing imaging techniques, image-based modelling approaches have proven to be a valuable method for investigating the control of branching events with respect to organ-specific properties.

Consequently, a pipeline has been established that allows to test models on physiological geometries from cultured embryonic kidney and lung explants (Adivarahan et al., 2013; Menshykau and Iber, 2013; Iber et al., 2015, 2016; Gómez et al., 2017). The obtained 2D time-lapse movies are segmented to obtain the epithelial boundary for each time frame and to calculate the growth fields between consecutive time frames. In order to solve models on these geometries, the extracted domains need to be meshed. The simulated signalling fields can then be compared to the calculated growth fields. Similarly, this pipeline has been applied to 3D reconstructions of lung explants of different embryonic stages (Menshykau et al., 2014).

## Ligand-Receptor-Based Turing Mechanism

The Turing mechanism has been suggested for many biological pattern phenomena, including morphogenesis (Miura and Maini, 2004; Kondo and Miura, 2010; Guo et al., 2014a,b; Xu et al., 2017), and is based on a diffusion-driven instability which leads to the self-organised emergence of many different kind of patterns (Turing, 1952; Gierer and Meinhardt, 1972). The underlying network structure includes at least two factors with substantially different diffusion rates that interact in a cooperative way leading to the upregulation of one of the factors. These requirements are typically true for many ligand-receptor systems.

**FIGURE 2:**
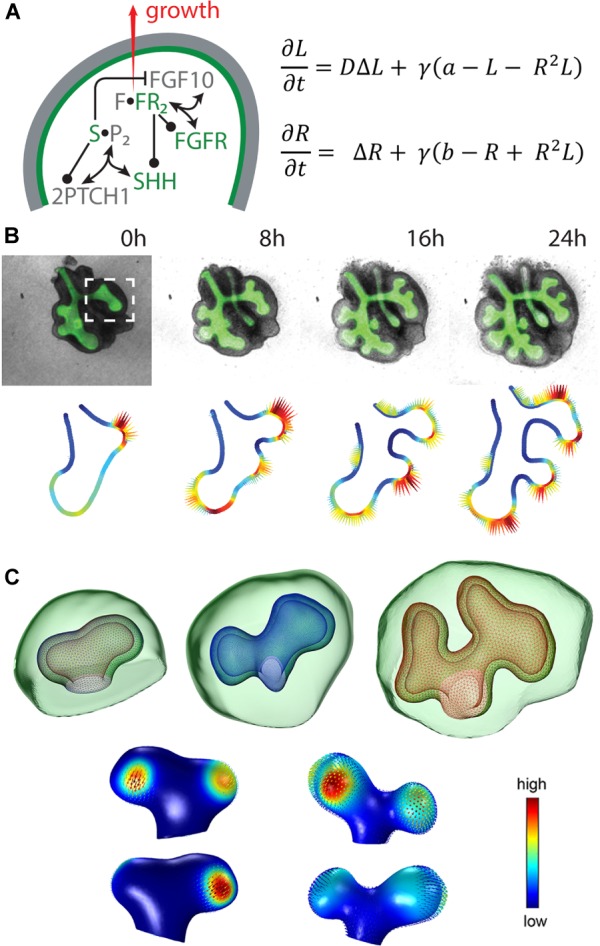
The ligand-receptor based Turing model reproduces lung bud outgrowth in 2D and 3D. (**A**, left) Signalling network in lung branching morphogenesis includes the interactions between FGF10, SHH and the corresponding receptors in the epithelium (green) and the mesenchyme (grey). Reproduced with permission from Iber and Menshykau (2013). (**A**, right) The diffusion-reaction equations for ligand (L) and receptor (R) consider the diffusion coefficient ratio D, a scaling factor γ, production rates a and b, linear decay as well as cooperative binding. (**B**, top) 2D time-lapse data of an embryonic mouse lung showing the EGFP-expressing epithelium (green) and the mesenchyme (grey). Reproduced with permission from Menshykau et al. (2014). (**C**, top) 3D sequence of a mouse lung bud with epithelium (wireframe) and mesenchyme (green). Reproduced from Menshykau et al. (2014). (**B,C**, bottom) The predicted signalling strength (solid colour) matches the experimentally observed growth fields (vector fields) in 2D and 3D, respectively. Reproduced with permission from Menshykau et al. (2014).

For lung branching morphogenesis, a reaction-diffusion model has been proposed that includes FGF10 and sonic hedgehog (SHH) as key signalling factors (Figure 2A) (Celliere et al., 2012; Menshykau et al., 2012). In order to examine whether the Turing mechanism can be extended to other branched organs, the ligand-receptor Turing model has been applied to kidney branching morphogenesis by considering the regulatory interactions between GDNF and WNT11 (Adivarahan et al., 2013; Menshykau and Iber, 2013; Menshykau et al., unpublished).

Solving the model on static idealised 2D and 3D domains shows that the described interactions give rise to Turing patterns that correspond to FGF10 or GDNF signalling patterns, respectively, representing the branching modes of lateral branching, bifurcations and trifurcations (Menshykau et al., 2012, 2014; Menshykau and Iber, 2013). As reported for embryonic kidney development, in this model bifurcations and trifurcations dominate over bud elongation and lateral branching, while trifurcations do not appear for the lung. Moreover, the model reproduces not only wild type but also mutant data (Menshykau et al., 2012).

Taking into account the dynamical processes during branching morphogenesis, 2D time-lapse movies of cultured lung and kidney explants and a 3D sequence of lung buds have been used to test alternative models and showed that only the ligand-receptor Turing model reproduces the areas of outgrowth for these physiological geometries (Figures 2B,C) (Menshykau et al., 2014; Menshykau et al., unpublished). Solving the model on 3D growing domains confirmed that the predicted signalling patterns support actual outgrowth (Menshykau et al., 2014).

The emerging Turing pattern is typically very dependent on the initial conditions. Combining tissue-specific expression of the considered signalling factors with the ligand-receptor based Turing model allowed for robust outgrowth behaviour despite noisy initial conditions due to the impact of the geometry (Menshykau et al., 2014; Menshykau et al., unpublished).

Therefore, the ligand-receptor based Turing mechanism potentially constitutes a common mechanism for regulating branching morphogenesis in both lungs and kidneys. However, Turing patterns only explain the branch point selection but not the regulation of branch lengths, widths or angles. In the lung, the length and width of branches seems to be controlled by a bias in cell division (Tang et al., 2011). Moreover, the Turing mechanism is highly sensitive to the included interactions and choice of parameter values and can only be applied in a qualitative but not in a quantitative manner (Combes, 2015).

## Concluding Remarks

In this review, we focused on the signalling interactions during lung and kidney morphogenesis and discussed several modelling approaches. Based on the robustness and flexible applicability to different branched organs, we conclude that the ligand-receptor based Turing mechanism represents a potential candidate for a general regulatory mechanism for branching morphogenesis. However, several studies exist confirming that mechanical stresses influence branching morphogenesis (Lubkin and Murray, 1995; Lubkin, 2008; Unbekandt et al., 2008; Wan et al., 2008; Gjorevski and Nelson, 2010, 2012; Nelson and Gleghorn, 2012; Kim et al., 2013; Varner and Nelson, 2014). Since there is a close interaction between signalling factors and mechanical tissue properties, modelling approaches should also take into account the mechanics behind branching morphogenesis (Tanaka, 2015; Peters and Iber, 2017).

Although mathematical models have advanced our understanding of branching morphogenesis, none have yet proven to fulfil all criteria to explain the branching behaviour on a holistic level. This may be attributed to a lack of biological methods to obtain quantitative information on relevant modelling parameters and computational limitations regarding analysis of large imaging data. The latter is also relevant for solving complex mathematical models, which requires tremendous computational resources. However, combining the constant improvements in all domains will improve the conclusions that can be drawn from modelling approaches. We are hopeful that data based modelling will continue to improve our understanding of branching morphogenesis.

## Author Contributions

All authors listed have made a substantial, direct and intellectual contribution to the work, and approved it for publication.

## Conflict of Interest Statement

The authors declare that the research was conducted in the absence of any commercial or financial relationships that could be construed as a potential conflict of interest.
